# Influenza infection directly alters innate IL-23 and IL-12p70 and subsequent IL-17A and IFN-γ responses to pneumococcus in vitro in human monocytes

**DOI:** 10.1371/journal.pone.0203521

**Published:** 2018-09-07

**Authors:** Sinead T. Loughran, Patrick A. Power, Paula T. Maguire, Samantha L. McQuaid, Paul J. Buchanan, Ingileif Jonsdottir, Robert W. Newman, Ruth Harvey, Patricia A. Johnson

**Affiliations:** 1 Viral Immunology Laboratory, School of Nursing and Human Sciences, Dublin City University, Dublin, Ireland; 2 Translational Cancer Physiology Laboratory, School of Nursing and Human Sciences, Dublin City University, Dublin, Ireland; 3 Department of Immunology, Landspitali, The National University Hospital of Iceland, Reykjavik, Iceland; 4 National Institute for Biological Standards and Controls, Potters Bar, Herts, United Kingdom; Instituto Butantan, BRAZIL

## Abstract

It is well accepted that influenza A virus predisposes individuals to often more severe superinfections with *Streptococcus pneumonia*. However, the mechanisms that lead to this synergy are not clearly understood. Recent data suggests that competent Th17 immunity is crucial to clearance and protection from invasive pneumococcal disease of the lung. We demonstrate that early influenza infection significantly reduced levels of pneumococcus driven IL-12p70, IL-23 and IL-27 in human monocytes with significant impairment of IL-17A and IFN-γ in HKSP-treated allogeneic mixed lymphocyte cultures. We also provide evidence to suggest that the hemagglutinin component of the virus is at least partially responsible for this downward pressure on IL-17 responses but surprisingly this suppression occurs despite robust IL-23 levels in hemagglutinin-treated monocyte cultures. This study demonstrates that influenza can directly affect the immunological pathways that promote appropriate responses to *Streptococcus pneumonia* in human immune cells.

**Importance:**

Influenza virus is highly contagious and poses substantial public health problems due to its strong association with morbidity and mortality. Approximately 250,000–500,000 deaths are caused by seasonal influenza virus annually, and this figure increases during periods of pandemic infections. Most of these deaths are due to secondary bacterial pneumonia. Influenza-bacterial superinfection can result in hospitalisation and/or death of both patients with pre-existing lung disease or previously healthy individuals. The importance of our research is in determining that influenza and its component haemagglutinin has a direct effect on the classic pneumococcus induced pathways to IL-17A in our human ex vivo model.

Our understanding of the mechanism which leaves people exposed to influenza infection during superinfection remain unresolved. This paper demonstrates that early infection of monocytes inhibits an arm of immunity crucial to bacterial clearance. Understanding this mechanism may provide alternative interventions in the case of superinfection with antimicrobial resistant strains of bacteria.

## Introduction

The devastating synergism of bacterial pneumonia with influenza viral infections left its mark on the world over the last century. Although the details of pathogenesis remain unclear, the synergism is related to a variety of factors including pulmonary epithelial barrier damage which exposes receptors that influence bacterial adherence and the triggering of an exaggerated innate immune response and cytokine storm, which further acts to worsen the injury. Several therapeutics and combination therapies of antibiotics, anti-inflammatories including corticosteroids and toll-like receptor modifiers, and anti-virals are being discussed. This mini review summarizes recent developments in unearthing the pathogenesis of the lethal synergism of pneumococcal co-infection following influenza, as well as addresses potential therapeutic options and combinations of therapies currently being evaluated.

There is considerable evidence from animal models, and clinical data from humans that Influenza A virus (IAV) predisposes individuals to bacterial infection typically with capsular, extracellular bacteria such as *Streptococcus pneumoniae* (*S*.*p*) and *Staphylococcus aureus* [1–3]. Influenza-bacterial superinfection can result in hospitalisation and/or death of both patients with pre-existing lung disease or previously healthy individuals [4,5]. In the 1918 pandemic, the late 1960’s pandemic, and the 2009 pandemic, the predominant bacterial co-pathogen was *Streptococcus pneumoniae* [6–8]. Pandemics are not the only threat however, as approximately one million deaths are caused by seasonal influenza virus annually [6,9], and most of these deaths are due to secondary bacterial pneumonia [10]. In seasonal IAV, the most common co-infecting bacteria is *Staphylococcus aureus* in adults, however in children, globally it is *Streptococcus pneumoniae* [10], whilst in the U.S., *Staphylococcus aureus* has been implicated [11,12]. In addition, an important clinical manifestation of particular concern is the increase in antibiotic resistant strains of bacteria [2]. Understanding of the immunological mechanisms that trigger susceptibility to bacterial disease during IAV infection may provide more treatment strategies, particularly for superinfection with antibiotic resistant strains.

Although considerable progress has been made over the last decade, no real consensus has been reached. Earlier studies have suggested that physiological damage to the respiratory epithelium, along with increased adherence factors for *S*.*p* may be involved [1]. While these processes are likely to contribute to more enhanced colonisation, recent research has pointed to the role of immunological mechanisms in the susceptibility to invasive bacterial disease during influenza infection [1,4,13,14]. A strong indication for a role of impaired immunological responses have been suggested in mice such as reduced responsiveness of alveolar macrophages [15], and elevated levels of anti-inflammatory IL-10 [14,16]. Also, a neutrophil influx caused by viral and bacterial toxins result in a cytokine storm leading to a destructive hyper inflammatory response in mice [17].

Recently, Th17 cells have been identified as critical in the effective clearance of extracellular capsular bacteria from the lung and further studies have revealed that IAV infection has been shown to inhibit the Th17 response in mice [2]. Th17 cells are distinct from Th1 cells, which control intracellular bacterial infections [18]. Th17 cells are the primary producers of interleukin-17 (IL-17, also known as IL-17A), a pro-inflammatory cytokine [18], with a specific role in the recruitment of neutrophils and macrophages [13]. It is well known that the cytokines, transforming growth factor-β (TGF-β), IL-6 and IL-1β drive Th17 differentiation, while IL-23 conserves the expansion [19] and commitment to the Th17 lineage [20], thus increasing the production of IL-17A [19]. Late viral induction of Type I and Type II interferons have been shown to inhibit these essential Th17 responses to pneumococcus in mice [2,21]. Type I interferons include the subtypes IFN-α, of which there are thirteen distinct proteins, and IFN-β, of which there is only a single protein [22]. Type I IFNs provide anti-viral protection by inhibiting influenza replication and directing Th1 adaptive immunity [23].

A study found that the antiviral response of Type I IFNs predisposed mice to secondary infections with *S*.*p*. Mice without functional IFN-α/β receptor (IFNAR) signalling were more resistant to secondary bacterial pneumonia than mice with functional IFNAR signalling. Also, mice deficient in type I IFN signalling are less susceptible than wild type mice to *S*.*p*. super–infection at day 7 after influenza infection [2]. However, in contrast to these reports, it has been shown that IFN-α expression prior to respiratory infection with *S*.*p*. improved the outcome of pneumococcal infection in mice [24], and that IFNAR signalling can be crucial for *S*.*p*. bacterial clearance [22,25–28]. There are also conflicting reports as to the effects of IFN-γ (Type II IFN) during *Streptococcus pneumoniae* infection in mice. It has been shown that IFN-γ did not have a protective role against *Streptococcus pneumoniae*, as IFN-γ receptor-deficient (IFN-γR^-/-^) mice were found to have significantly fewer pneumococci in their lungs than wild-type mice, and IFN-γ^-/-^ mice had fewer colony-forming units present in the lungs than wild-type mice [29]. Another study found that in the presence of an influenza infection, IFN-γ produced in the lung by T-cells inhibited macrophage-mediated bacterial clearance, which is essential in the clearance of pneumococci, resulting in an increase in susceptibility to secondary bacterial infections [3]. In contrast to these studies, it has been shown that IL-12 and IFN-γ can mediate protective effects against *Streptococcus pneumoniae* by promoting neutrophil accumulation [30] and that IFN-γ produced by neutrophils during *Streptococcus pneumoniae* infection was important in host defence as mice deficient in IFN-γ had impaired bacterial clearance [31].

There have been significant studies in mice, but there is a need to add sufficiently to studies in human models so that this complex relationship can be fully explored. Much of this work has focused on the indirect effect of influenza host immune response to infection on the impaired development of immune protection to bacterial disease. Our group has published data which demonstrates that hemagglutinin (HA) from influenza virus directly down-regulates bacterial LPS induced IL-12p35 in mice [4]. Although gram negative bacteria have been associated with superinfection with influenza, the overwhelming evidence points to susceptibility to gram-positive capsular bacteria such as S.p. during influenza infection [9]. In this study, we therefore investigated if IAV infection or components specific to IAV, in addition to anti-viral host responses could alter appropriate immunity to gram-positive pneumococcus in humans. We also sought to investigate if immunomodulation was strain specific and we examined the responses in humans to H1N1 and H3N2 viruses.

In order to model the environment generated by influenza infection of the lung, in a previous study we comprehensively compared the maturation profiles (phenotypic expression, cytokine production, apoptosis, and T cell proliferation) of untreated monocytes, IL-4-treated dendritic cells (DCs), IFN-treated DCs, and DCs following co-culture with supernatant from influenza infected lung epithelial cells. We found that direct influenza infection of monocytes and monocytes cultured with supernatants from virally infected lung epithelial cells induce distinct DC subsets compared with influenza infection of artificially generated IL-4-treated DCs and IFN-treated DCs. We concluded that artificially generated DCs skewed immune responses to influenza infection, whereas direct influenza infection of monocytes mimic those generated from influenza infected epithelial cells supernatants and appear to more accurately mimic those generated in vivo [32].

This model is based on comprehensive analysis of the signals provided by influenza infected lung epithelial cells which will influence the extravasation and maturation of monocytes from the periphery [32]. Monocytes directly infected by influenza are referred to as APCs. Thus, we chose to infect primary human monocytes isolated from healthy volunteers directly with influenza and assess the impact these infections have on innate responses to heat killed *S*.*p* in human APCs. To ascertain the adaptive responses, a mixed lymphocyte reaction (MLR) was used, which is commonly used in the investigation of T cell responses [4,32–38]. In this assay, infected/treated APCs from one donor were co-cultured with human primary T cells from a different donor resulting in an allogeneic response due to the mismatched major histocompatibility complex (MHC) antigens. as per previous studies [4,32]. We show strong and consistent evidence that live infection with Influenza virus down-regulates innate cytokines in HKSP-treated cells such as IL-23 and IL-12p70 involved in Th17/Th1 generation. These responses occur without elevated innate anti-inflammatory cytokines such as IL-10 and TGF-β, with negligible IFNα/β protein production and with reduced IL-27 production.

We have further evidence to suggest that the HA component of IAV is at least partially responsible for this downward pressure on IL-17 responses. Surprisingly this suppression by HA occurs despite robust levels of IL-23 in HA treated APC cultures. This direct effect on the classic IL-17A pathway by IAV and its components over and above the late induction of type-I and type-II IFNs may further explain the overwhelming synergy between influenza and extracellular capsular bacteria.

## Materials and methods

### Ethical statement

Ethical approval for this study was granted by Dublin City University’s Research Ethics Committee.

### Separation of peripheral blood mononuclear cells (PBMCs)

Buffy coats from healthy donors were obtained from the Irish Blood Transfusion service (at St James’ hospital, Dublin). The peripheral venous blood (approximately 50 ml) was mixed with 5 ml of a 5% solution of EDTA and diluted 1:2 with HBSS containing 1% FBS, and 10 μM HEPES buffer. This diluted blood was layered onto 14 ml of density gradient medium Lymphoprep^TM^ (Axis-shield, Norway) and centrifuged at 400 x g for 25 minutes (with accelerator and break switched off). With blood components separated according to density, the buffy coat layer was removed and cells were washed twice with 10 ml of complete RPMI (cRPMI) (supplemented with 10% FBS, 10 mM HEPES, and 100U penicillin/ml). Finally, cells were filtered through a 40 μM filter and diluted to required cell number per ml.

### Separation, purification and depletion of cell subsets from PBMCs using microbead separation

Cells were purified from PBMCs using magnetic microbeads by MACS (Miltenyi Biotec, UK). PBMCs were resuspended in MACs buffer (sterile PBS supplemented with 0.5% bovine serum albumin (BSA) and 2 μM EDTA) and incubated with target-cell specific antibodies conjugated to magnetic microbeads according to the manufacturer’s instructions. Following incubation, cells were washed with MACs buffer. For positive selection of CD14^+^ or CD3^+^ cells, cell suspension was applied to an LS column attached to a magnet. The column was washed 3 times with 3 ml of MACs buffer. The positively labelled cells were flushed out of the column with a plunger in the absence of magnetic force. The initial “flow through” and wash step were used as the CD14^+^ cell depleted fraction. CD14^+^ and CD3^+^ cells were isolated from different donors. CD3^+^ T cells at a density of 2x10^5^ cells/ml were co-cultured with 10^6^ cells/ml of treated CD14^+^ cells and incubated for a further 24 h at 37°C and 5% CO_2_.

### Viruses and infection experiments

Isolated CD14^+^ cells at a density of 10^6^ cells/ml were exposed to *Heat Killed S*.*p (*HKSP) serotype Ι (ATCC #6301, VA, USA), (10^7^ CFU) (cultured as below) or Heat Killed Streptococcus pneumoniae (HKSP) (10^7^ CFU) (Invivogen), live H1N1 (Puerto-Rico/8/34) or H3N2 (Wisconsin/67/2005, A/Panama/2007/99 NIBSC, UK, and A/Uruguay/716/2007), (4.9 PFU/ml), H1N1 HA (Puerto-Rico/8/34) or H3N2 HA (A/Wisconsin/67/2005 and A/Uruguay/716/2007) (1 μg ml^-1^ or 3 μg ml^-1^), alone or in combination with HKSP for 24 h.

### Culture of *Streptococcus pneumoniae*

Lyophilised *Streptococcus pneumoniae* (ATCC #6301, serotype I) pellet was resuscitated with 1 ml of brain heart infusion broth (BHIB) and transferred to 100 ml BHIB for overnight culture at 37°C. Streak plates were prepared and gram positive cocci were confirmed by gram stain. The culture was incubated until OD^600^ 0.633, after which it was centrifuged at 3,000 x g for 10 min, washed three times in sterile pyrogen-free PBS and resuspended in 50 ml PBS. The number of bacteria in the final suspension was determined by plating 10-fold serial dilutions onto agar plates. Heat inactivation was accomplished in a water bath at 60°C for 90 min. Streak plates were prepared and no live bacteria were detected after the heat killed *Streptococcus pneumoniae* suspension was plated and incubated overnight. Aliquots of pneumococci were prepared and kept frozen at −70°C.

### Poly(I:C) stimulations

Poly(I:C) (1 μg/ml) was mixed with 100 ul LyoVec^TM^ solution and incubated at RT for at least 15 minutes. Isolated CD14^+^ cells at a density of 10^6^ cells/ml were stimulated Poly(I:C) and LyoVec^TM^ solution (Invivogen) and incubated for 24 h at 37°C.

### qPCR

Total RNA was isolated by using either the RNeasy mini kit (Qiagen), including DNase digestion (RNase-free DNase kit; Qiagen) or the RNeasy Plus mini kit (Qiagen). Total RNA was reverse transcribed into cDNA (GoScript Reverse Transcription System; Promega). cDNA samples were amplified using the Lightcycler Nano (Roche Diagnostics) and Faststart Essential DNA Probes Master System (Roche Diagnostics) with RealTime Ready Assays (Roche Diagnostics). Expression of IFN-α (Assay ID: 145795), IFN-β (Assay ID: 145797), and GAPDH. The chosen IFN-α probes detect a sequence which is common to all IFN-α subtypes. Gene expression was normalised to expression of the reference gene GAPDH.

### Enzyme-linked immunosorbent assay

Supernatant from treated cells (as above) was used to detect for the following cytokines using ELISA kits; IL-23, IL-12p70 (eBioscience/Biosciences), IL-6, IL-1β, IL-27, TGF-β, IL-10, IFN-α, and IFN-β (R&D systems) were used to quantify cytokine levels in supernatants according to manufacturer’s protocol. At least 3 experimental repeats of each treatment were analysed for each cytokine/donor.

### Apoptosis studies

Treated CD14+ cells were stained with annexin V Apoptosis Detection Kit I (BD-Pharmingen) according to manufacturer’s protocol and detected using FACSCalibur^TM^ Flow Cytometer/CellQuest software (BD-Biosciences). CD14^+^ cells treated for 24 h as outlined above, were dual stained with FITC annexin V and propidium iodide. The percentages of viable, necrotic/late apoptotic, early apoptotic cells after treatments were ascertained using flow cytometry for 3 independent donors.

### Western blot

Treated cells were lysed for 10 min on ice in suspension buffer (0.1 M NaCl, 0.01 M Tris-Cl (pH 7.6), 0.001 M EDTA (pH 8.0), 1 μg/ml leupeptin, 1 μg/ml aprotinin, 100 μg/ml PMSF). An equal volume of 2 X SDS gel loading buffer (100 mM Tris-Cl (pH 7.6), 4% (w/v) SDS, 20% (w/v) glycerol, 10% (v/v) 2-mercaptoethanol 0.2% bromophenol blue) was immediately added to the cell suspension followed by sonication for 20 s with 1-s pulses (40/output, 200 W). The lysate was clarified by centrifugation at 12,000 x g for 10 minutes at room temperature followed by boiling at 95°C for 5 min. Approximately equal numbers of cell equivalents were loaded and SDS-PAGE was performed using 10% (v/v) resolving gels and 5% (v/v) stacking polyacrlyamide gels. Proteins were transferred to 0.45 μm nitrocellulose membranes. Membranes were blocked in TBS+5% milk for 1 h at room temperature, then probed with Mouse Anti-human-β-actin clone AC-15 (Sigma-Aldrich) in TBS+5% milk for 2 h at room temperature or overnight at 4°C. Membranes were washed and incubated with Goat anti-mouse IgG, Alkaline Phosphatase conjugated antibody (Promega) in TBS+5% milk for 1 h at room temperature. Membranes were washed and incubated in BCIP (Sigma-Aldrich) reagent for 5 min at room temperature and photographed.

### Statistical analysis

Statistical analyses were performed using GraphPad Prism version 6.0 for windows and Mac (GraphPad Software). Where stated, data was normalised by setting untreated sample readings to ~1 and comparing treated sample readings to that value, thus providing relative concentrations. Data has been normalised to allow for the presentation of data for multiple human donors; this is due to donor-to-donor variability in relation to actual concentration of cytokines produced. A One-way ANOVA was fitted to the data and comparisons of interest were made using a Sidak test to adjust for multiple testing, using a 5% significance interval; p-values less than 0.05 were considered significant and are represented as follows: *p<0.05, **p<0.01, ***p<0.001, ****p<0.0001.

## Results

### Influenza A virus infection inhibits innate immune responses in human APCs treated with HKSP

**Fig 1:**
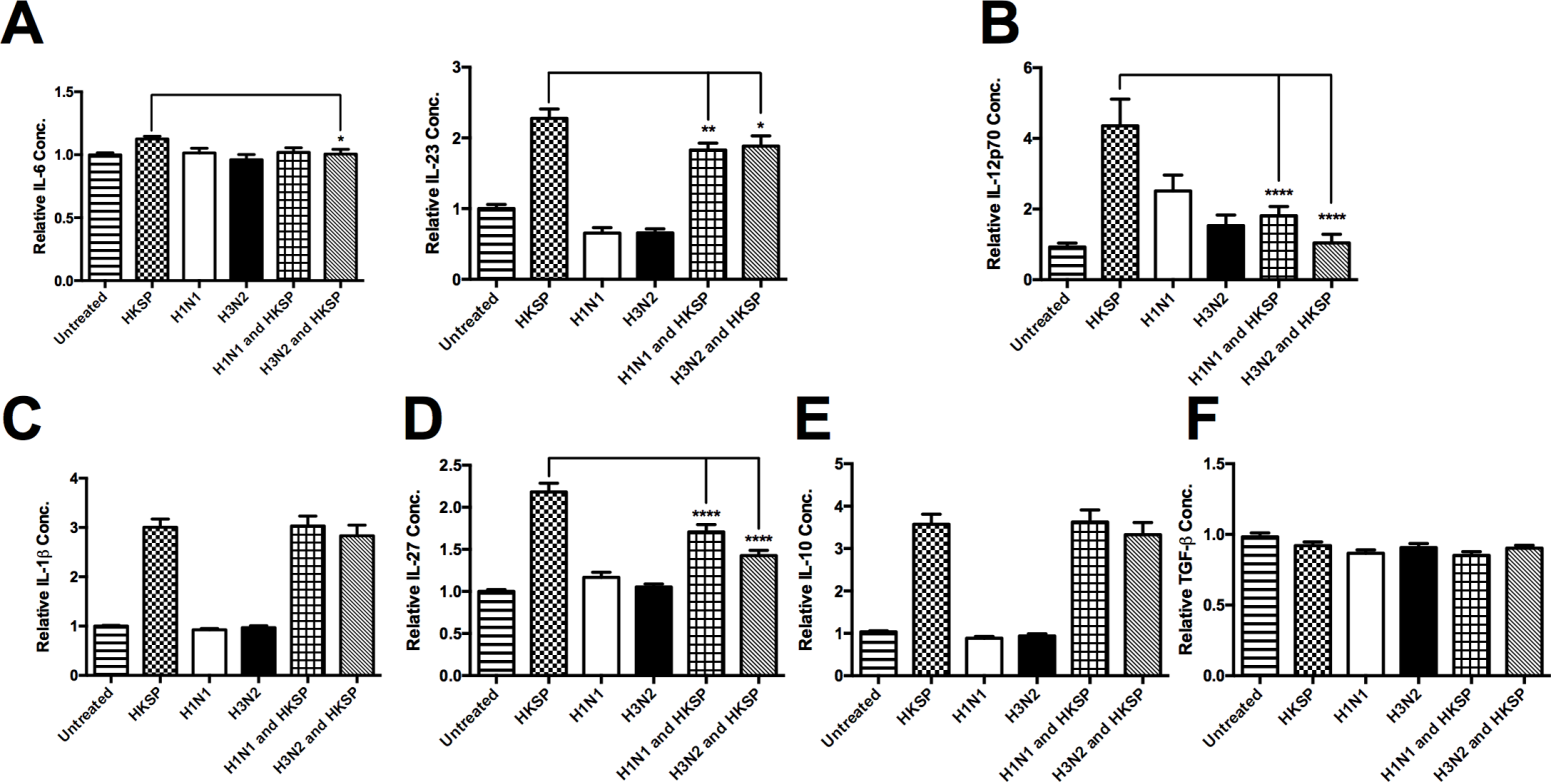
Live IAV attenuates innate cytokines in HKSP-treated cells. The levels of (A) IL-6, IL-23, (B) IL-12p70, (C) IL-1 β, (D) IL-27, (E) IL-10, and (F) TGF-β, secreted by CD14^+^ cells following 24 h treatment with HKSP, live H1N1 or H3N2 alone or in combination with HKSP (or untreated as a control) were determined by ELISA. Each column represents normalised mean cytokine levels + SEM of at least 3 experimental repeats of each treatment/donor. Each ELISA represents normalised results from multiple donors: (A) IL-6 (n = 5), IL-23 (n = 7), (B) IL-12p70 (n = 12), (C) IL-1 β (n = 11), (D) IL-27 (n = 10), (E) IL-10 (n = 9), and (F) TGF-β (n = 8). Statistical analyses were performed to compare cytokine levels in cells exposed to HKSP versus cells exposed to live H1N1 or H3N2 in combination with HKSP by fitting a One-way ANOVA to the data and using a Sidak test to adjust for multiple testing (*p<0.05, **p<0.01, ***p<0.001, ****p<0.0001).

Experimental animal models and clinical data from humans strongly support IAV as a predisposing agent to more severe bacterial infection with *S*.*p* [1]. Reports have suggested that late antiviral immunity while important in IAV clearance, can promote increased susceptibility to secondary bacterial infection [2,3]. However, this may not fully explain the remarkable specific synergy between IAV and superseding capsular, gram-positive bacteria. We separated peripheral blood mononuclear cells (PBMCs) from buffy coats from healthy human donors and have demonstrated that IAV infection inhibits innate responses in human APCs treated with HKSP 24 hours post-infection. We found that the H3N2 subtype of IAV significantly inhibited levels of IL-6 in pneumococcus-treated primary human monocytes (Fig 1A) (n = 5). Two subtypes of IAV (H1N1 and H3N2) significantly inhibited pneumococcus driven Th17 polarizing IL-23 (n = 7) (Fig 1A) and the Th1 polarizing IL-12p70 (n = 12) (Fig 1B). This reduction was not observed for the Th17 inducing cytokine, IL-1β (n = 11) (Fig 1C). We also found that IL-27 (n = 10) was induced by pneumococcus and this response was inhibited by IAV (Fig 1D). A reduction was not observed for the anti-inflammatory cytokine, IL-10 (n = 9) (Fig 1E), which has been shown to inhibit Th17 responses [39], or the multi-functional cytokine, TGF-β (n = 8) (Fig 1F).

### Influenza A virus significantly inhibits Th17/Th1 innate polarizing cytokines despite negligible or undetectable IFN-α or IFN-β message RNA or protein

**Fig 2:**
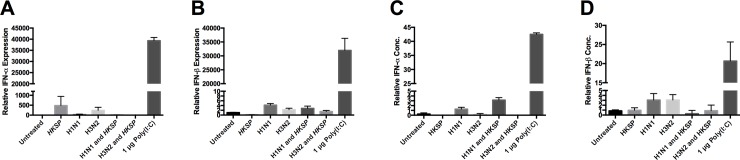
Modulation by IAV of innate and adaptive responses to Pneumococcus is not due to Type I IFN production. (A) IFN-α (n = 4) and (B) IFN-β (n = 3) mRNA production in CD14^+^ cells following 24 h treatment with HKSP, live H1N1 or H3N2 alone or in combination with HKSP (or untreated as a control), or 1 μg Poly(I:C) transfected with LyoVec solution. mRNA expression of IFN-α, IFN-β, and GAPDH from infected and control treatments was determined by qPCR. The level of IFN-α (n = 3) (C) and IFN- β (n = 3) (D) protein secreted by CD14^+^ cells following 24 h treatment with HKSP, live H1N1, H3N2 alone or in combination with HKSP or Poly(I:C) (or untreated as a control) were determined by ELISA.

Much of the previous studies in mice show that it is late viral Type I interferons that are responsible for inhibition of essential Th17 responses [2,21]. To investigate if the downward pressure on polarizing cytokines was due to the production of Type I IFNs, we used qPCR to detect for the presence of IFN-α and IFN-β message. In humans, there are 13 different IFN-α subtypes, to ensure capture of all subtypes, we implemented rigorous bioinformatics searches to ensure chosen probes and primers amplified a region of the mRNA sequence which was common to all subtypes. IFN-α was undetectable in three out of four donors, with very low transcription levels detectable in just one donor, compared to samples treated with Poly(I:C) (n = 4) (Fig 2A). Very low levels of IFN-β amplification was detected in all samples compared with samples treated with Poly(I:C) (Fig 2B). To establish the protein levels of IFN-α and IFN-β in supernatants, we used ELISA. IFN-α and IFN-β were induced strongly in Poly(I:C) treated cells compared to other cells (Fig 2C and 2D). IFN-α was detectable in all cells, apart from those treated with HKSP, and co-treated with H3N2 and HKSP (Fig 2C). IFN-β was detectable in very low amounts in all cells (Fig 2D). IFN-α has been found to be produced 3 days post-influenza infection, peaking at day 5 in mice [2]. The reason for such late anti-viral IFN responses has been attributed to the influenza viral protein, non-structural protein 1 (NS1), which contributes to the evasion of the hosts innate immune responses by blocking NF-κB, IRF3, and IRF7 activation, resulting in the inhibition of Type I IFNs [40]. In the absence of NS1, IFN-β mRNA message has been detected as early as 3 hrs post-infection [41], in addition it has previously been shown that baseline IFN-β protein levels are not sufficient to inhibit Th17 responses in humans [42].

### Inhibition of Th17/Th1 innate polarizing cytokines are not due to cell death or shutdown of protein synthesis

**Fig 3:**
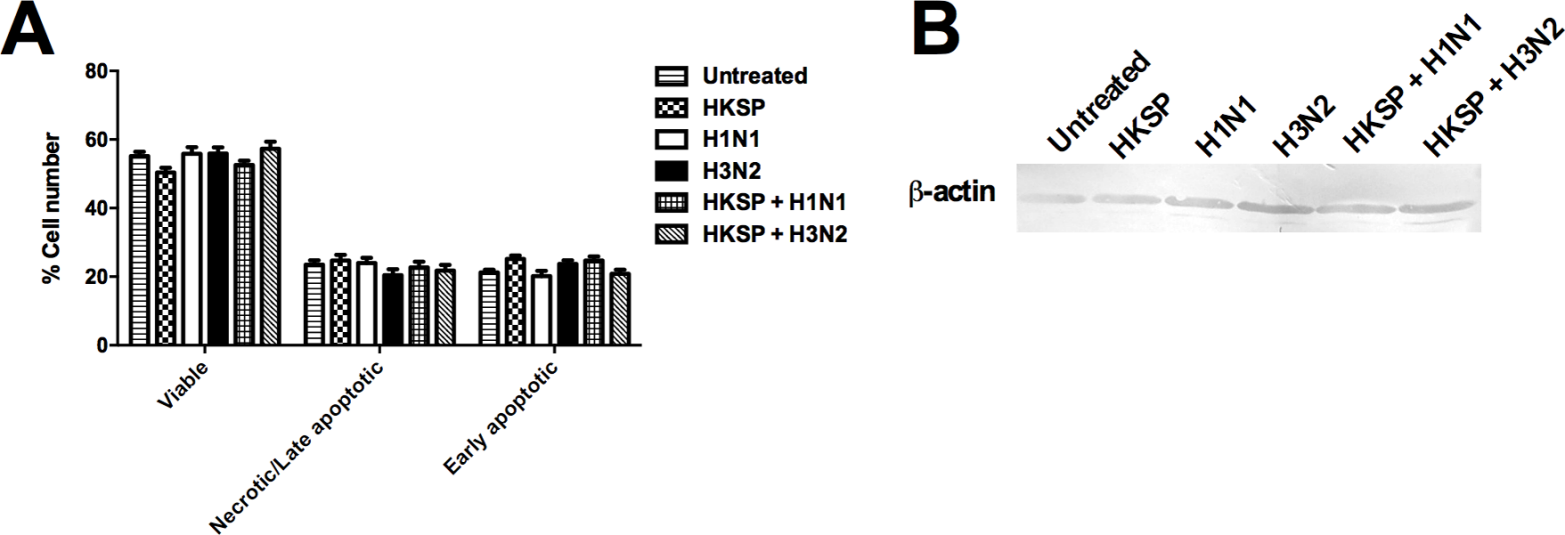
Modulation by IAV of innate and adaptive responses to Pneumococcus is not due to cell death or host protein synthesis shutdown. (A) CD14^+^ cells treated for 24 h with HKSP, live H1N1 or H3N2 alone or in combination with HKSP (or untreated as a control) were dual stained with FITC annexin V and propidium iodide. The percentages of viable, necrotic/late apoptotic, and early apoptotic cells after treatments were ascertained using flow cytometry. Each column represents mean % cell number + SEM of 3 independent donors. (B) Lysates from CD14^+^ cells treated for 24 h as outlined in (A) above, were prepared and analysed by Western blot for β-actin levels. Western blot is a representative of 2 independent repeats with different donors.

To ensure that the inhibition of innate Th17/Th1 polarising cytokines in human monocytes (APCs) is not due to cell death or protein synthesis shutdown, an apoptosis study and western blot were performed, respectively. The effects on the Th17 and Th1 cytokines were detected within 24 h of simultaneous co-culture and were not due to cell death (Fig 3A). Host protein synthesis shutdown did not occur as we detected the presence of the house-keeping protein, β-Actin (Fig 3B). This corresponds with the selective nature of the viral inhibition of cytokine levels. The crucial role for IL-17 in HKSP immunity is unquestionable [1,2]. Thus, it would appear, in this ex vivo human model, that influenza infection directly affects immune pathways important to the resolution of HKSP infection which is not due to cell death or protein synthesis shutdown.

### Influenza A virus inhibits polarisation of human allogeneic T cells towards Th17 in HKSP-treated cells

**Fig 4:**
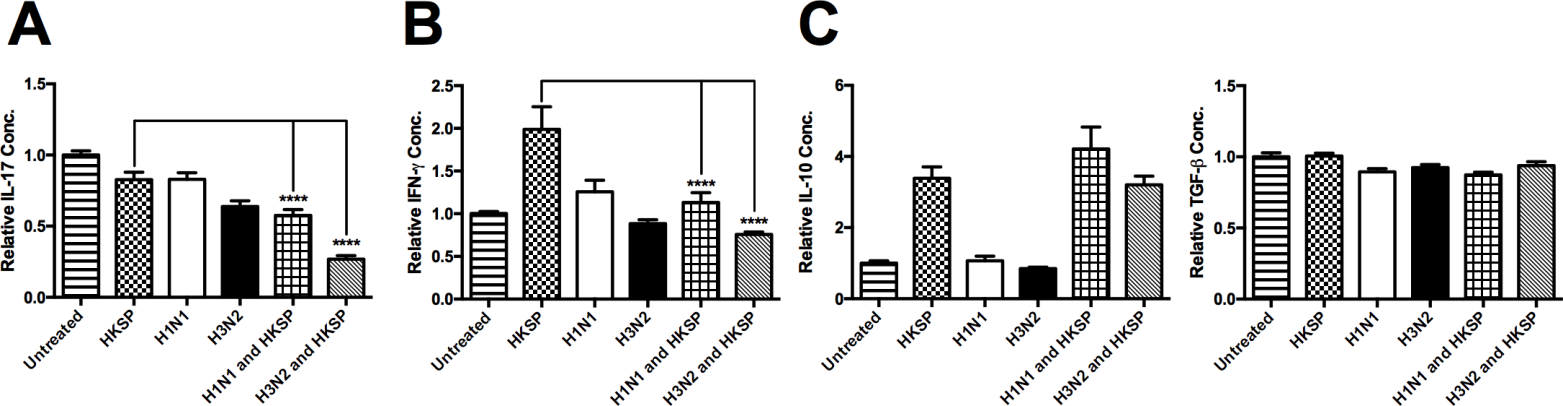
Live IAV attenuates adaptive cytokines in pneumococcus-treated cells. (A) IL-17A, (B) IFN-γ, (C) IL-10, and TGF-β, levels induced in allogeneic CD3^+^ T cells, co-cultured with CD14^+^ cells following 24 h treatment with HKSP, live H1N1 or H3N2 alone or in combination with HKSP (or untreated as a control) were determined by ELISA. Each column represents normalised mean cytokine levels + SEM of at least 3 experimental repeats of each treatment/donor. Each ELISA represents normalised results from multiple donors: (A) IL-17A (n = 9), (B) IFN-γ (n = 13), (C) IL-10 and TGF-β (n = 3). Statistical analyses were performed to compare cytokine levels in cells exposed to HKSP versus cells exposed to live H1N1 or H3N2 in combination with HKSP by fitting a One-way ANOVA to the data and using a Sidak test to adjust for multiple testing (*p<0.05, **p<0.01, ***p<0.001, ****p<0.0001).

To investigate the adaptive response, the use of allogeneic APC-T cell co-cultures was implemented as described before [4,32]. The allogeneic APC-T cell co-cultures also demonstrated that IAV infection had a profound effect on IL-17A production in response to HKSP treatment (n = 9) and IFN-γ (n = 13) (Fig 4A and 4B) indicating impaired Th17/Th1 responses in these co-cultures. Furthermore, the attenuation of the innate and adaptive IL-17 and IFN-γ pro-inflammatory pathways (Fig 4A and 4B) cannot be associated with increased innate expression of the anti-inflammatory cytokines IL-10 and TGF-β (n = 3) as no statistically significant increases were observed (Fig 4C). Although, a slight but nonsignificant increase of IL-10 was noted in cell supernatant co-treated with HKSP and H1N1, inhibition was also observed in cells co-treated with HKSP and H3N2 where no elevation of IL-10 was detected.

### Influenza HA selectively modulates human innate immune responses to pneumococcus treatment

**Fig 5:**
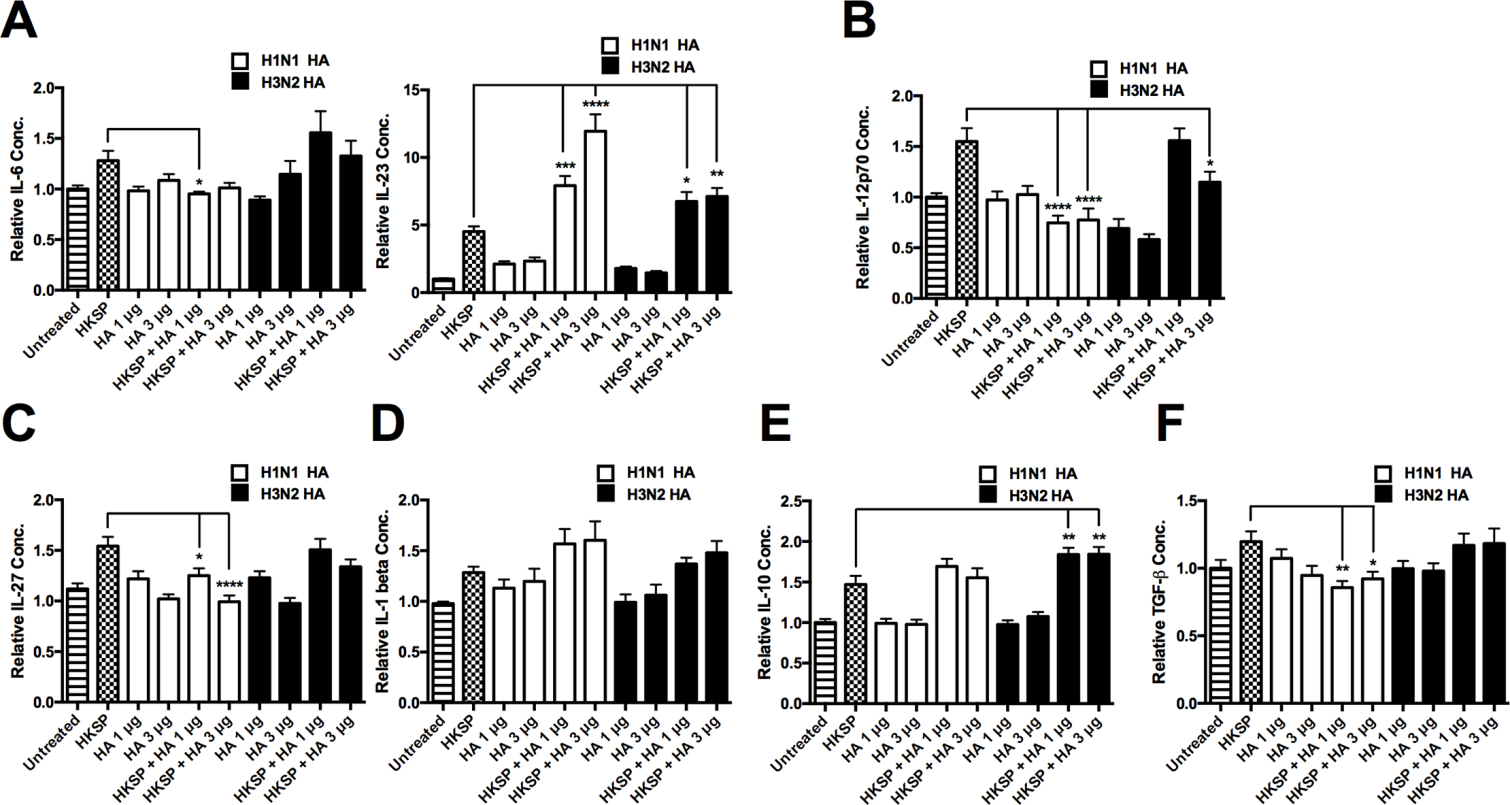
Influenza HA specifically attenuates innate cytokines in HKSP-treated cells. The levels of (A) IL-6, IL-23, (B) IL-12p70, (C) IL-27, (D) IL-1β, (E) IL-10 and (F) TGF-β secreted by CD14^+^ cells following 24 h treatment with HKSP, H1N1 HA (1μg ml^-1^ or 3 μg ml^-1^) or H3N2 HA (1μg ml^-1^ or 3 μg ml^-1^) alone or in combination with HKSP (or untreated as a control) were established by ELISA. Each column represents normalised mean cytokine levels + SEM of at least 3 experimental repeats of each treatment/donor. Each ELISA represents normalised results from multiple donors: (A) IL-6 (n = 3), IL-23 (n = 4), (B) IL-12p70 (n = 3), (C) IL-27 (n = 6), (D) IL-1β (n = 4), (E) IL-10 (n = 4), and, TGF-β (n = 3). Statistical analyses were performed to compare cytokine levels in cells exposed to HKSP versus cells exposed to H1N1 HA or H3N2 HA (1μg ml^-1^ or 3 μg ml^-1^) in combination with heat-inactivated HKSP, by fitting a One-way ANOVA to the data and using a Sidak test to adjust for multiple testing (*p<0.05, **p<0.01, ***p<0.001, ****p<0.0001).

We had previously observed that influenza HA down regulated LPS induced IL-12p70 in mice and that subtype specific anti-HA could restore these responses in murine derived bone marrow-derived dendritic cells (BMDCs) [4]. We were interested in establishing if this component was involved in the suppression of pneumococcal responses by IAV in these human cultures. In contrast to live infection, H1N1 HA actually inhibited pneumococcus induced IL-6 (n = 3), whereas H3N2 HA did not (Fig 1A). Strikingly, HA actually induced robust levels of IL-23 (n = 4) (Fig 5A) when combined with HKSP. Similarly, to live infection, both subtypes of IAV HA significantly attenuated HKSP induced IL-12p70 (n = 3) (Fig 5B), with a significant down-regulation of pneumococcus induced IL-27 (n = 6) by H1N1 HA (Fig 5C). Also in contrast to live infection, HA enhanced levels of IL-1β (n = 4) (Fig 5D) when combined with HKSP, although this was not statistically significant. Interestingly, H3N2 HA decreased levels of IL-10 (n = 4) (Fig 5E) and H1N1 HA increased levels of TGF-β (n = 3) (Fig 5F) in combination with HKSP.

### Influenza HA inhibits adaptive human responses to pneumococcus

**Fig 6:**
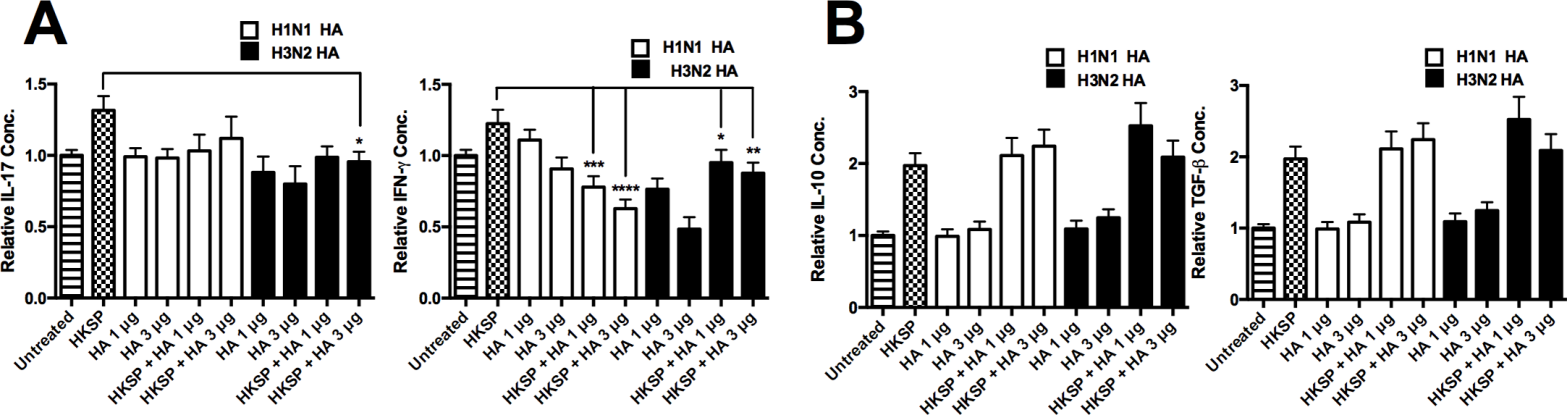
Influenza HA specifically attenuates adaptive cytokines in HKSP-treated cells. (A) IL-17A, IFN-γ, (B) IL-10 and TGF-β levels induced in allogeneic CD3^+^ T cells, co-cultured with CD14^+^ cells following 24 h treatment with HKSP, H1N1 HA (1μg ml^-1^ or 3 μg ml^-1^) or H3N2 HA (1μg ml^-1^ or 3 μg ml^-1^) alone or in combination with HKSP (or untreated as a control) were determined by ELISA. Each column represents normalised mean cytokine levels + SEM of at least 3 experimental repeats of each treatment/donor. Each ELISA represents normalised results from multiple donors: (A) IL-17A (n = 3), (B) IFN-γ (n = 3), (C) IL-10 and TGF-β (n = 6). Statistical analyses were performed to compare cytokine levels in cells exposed to HKSP versus cells exposed to H1N1 HA or H3N2 HA (1μg ml^-1^ or 3 μg ml^-1^) in combination with heat-inactivated HKSP, by fitting a One-way ANOVA to the data and using a Sidak test to adjust for multiple testing (*p<0.05, **p<0.01, ***p<0.001, ****p<0.0001).

Despite the significantly high levels of IL-23 and increased levels of IL-1β, we see a marked reduction in pneumococcus driven IL-17A and IFN-γ from Human allogeneic APC-T cell co-cultures when monocytes were primed with HA prior to co-culture. We see a reduction in pneumococcus driven IL-17A (n = 3) by H3N2 HA (3μg ml^-1^) and a reduction in pneumococcus driven IFN-γ (n = 3) by both H1N1 and H3N2 HA (Fig 6A). Unlike live infection, certain doses of HA were shown to increase the anti-inflammatory cytokines IL-10 and TGF-β (n = 6) (Fig 6B), although this was not statistically significant. However, these increases did not always correlate to doses of HA associated with down regulation of innate and adaptive IL-17 and IFN-γ pro-inflammatory pathways. The exclusion of an anti-inflammatory role is also supported by previous studies in mice where blocking IL-10 did not restore IL-12p70 levels in murine BMDCs [4]. These results suggest that HA may directly interfere with the IL-17 pathway without targeting the IL-23 or the IL-1β pathway.

## Discussion

Both clinical and animal data suggest that the greatest susceptibility to *S*.*p* infection occurs at the initiation of the recovery stage from IAV; approximately 7 days post influenza infection [1]. Researchers have therefore based their assumptions that it is the immune response to the virus occurring at this stage that contributes to the lethal synergy. This has been supported by several recent studies which suggest that the late type-I and type-II interferon response to the initial influenza infection render co-colonised hosts vulnerable to this lethal synergism [2,3]. However, during the 1918 pandemic, deaths were already occurring by day 7 [43]. While this antiviral response is likely to be an extremely important mechanism in influenza–pneumococcus co-pathogenesis, there may be other aspects of the interaction which could account for this synergism. Furthermore, this late anti-viral response may not be responsible for the increased susceptibility in children who have asymptomatic *S*.*p* carriage prior to influenza infection [44]. Recently, in an attempt to model what might occur in children, infant mice were colonised with *S*.*p* and subsequently infected with influenza. Within 6 days of IAV infection bacterial numbers had increased by 300-fold in the lung of co-infected animals [45], suggesting that synergistic mechanisms may also be occurring at earlier time points in infection. Additionally, an epidemiological study in humans of the incidence of bacterial co-infection in the 2009 influenza pandemic found that on average individuals developed co-infections, within the first 6.2 days of influenza infection. The authors suggest that co-infections coincide with high influenza viral shedding (2–3 days post-influenza infection), but may also occur simultaneously or shortly after influenza infection [46].

This is the first study to demonstrate an inhibitory effect of live IAV infection on IL-17A production in untreated allogeneic and pneumococcus-treated cells in humans. This study is also novel in that it identifies an inhibitory effect on IL-17A induction which is not dependent on type-Ι or type-II IFN production but involves at least one significant viral component. In cells co-treated with HKSP and IAV, we have observed no IFN-α and low levels of IFN-β message in qPCR analyses. Low levels of IFN-α protein were detected in cells co-treated with H1N1 and HKSP, but no IFN-α protein was detected in cells co-treated with H3N2 and HKSP. Levels of IFN-β protein was detected in cells co-treated with HKSP and IAV, however these were below levels in untreated cells. The presence of low levels of IFN-α protein in cells co-treated with HKSP and H1N1 cannot be responsible for the inhibition of HKSP responses as little to no IFN-α protein was detected in cells co-treated with HKSP and H3N2, where the same inhibition occurs. The presence of IFN-β protein cannot explain the attenuation of the Th1 and Th17 responses as these are below basal levels, which were present in all cells, including untreated and HKSP treated cells, in which we observed robust Th17 innate immune responses. In addition, a previous study in human cells demonstrated that basal levels of IFN-β are insufficient to inhibit Th17 responses [42]. Of note, we have observed that IAV inhibits pneumococcus-induced IL-27. IL-27 is a cytokine of much interest as it was shown to contribute to Th1 immunity by synergising with IL-12 in the production of IFN-γ [47]. However, more recent studies have shown that IL-27 has the ability to supress Th1, Th2, and Th17 responses in both humans and mice [42,48,49]. A more recent study in mice demonstrated that influenza-induced IL-27 led to a predisposition to secondary pneumococcal pneumonia due to suppression of IL-17A [41]. A similar result was also demonstrated in mice infected with influenza and *Staphylococcus aureus* [50]. In human cells, it was also observed that IL-27 inhibited IL-17A production and decreased the production of IL-23, IL-1β and IL-6 [41]. In contrast in our study, we observed a much stronger induction of IL-27 by HKSP than by IAV and an inhibition of IL-27 by IAV infection. These discrepancies may be due to the fact that we do not detect strong Type I IFN responses in IAV infected cells. It has been shown that IAV induces IL-27 in a IFNAR-dependent manner [41], which occurs with late-viral immune responses (32), subsequently, IL-27 can inhibit Th17 responses in a STAT1-dependent manner [51,52]. However, in the absence of STAT1, IL-27 has been shown to be involved in the differentiation of Th17 by inducing pSTAT3 in mice [53]. So, in the absence of Type I IFN signalling, IL-27 may enhance Th17 production. Indeed, it is possible that the reduction of HKSP induced IL-27 may contribute to the overall inhibition of the Th17 response. Due to the conflicting data regarding IL-27 and its role in the Th17 pathway, further studies are required in order to make robust conclusions regarding this cytokine. The inhibition of Th17 cytokines by IAV occurred in the absence of elevated levels of TGF-β, which can have either pro or anti-inflammatory effects [54,55]. TGF-β is neither induced by HKSP or inhibited by IAV; as TGF-β remains at basal levels despite treatment, TGF-β is not exerting any anti-inflammatory effects.

In our previous study, we demonstrated that IAV hemagglutinin inhibited lipopolysaccharide-induced IL-12p70 in murine derived bone marrow-derived dendritic cells [4]. In this study, we sought to investigate if hemagglutinin, a major component of IAV, which is also used in seasonal influenza vaccines, could be responsible for any of the inhibition observed in this human model. We found that although IAV inhibited HKSP induced IL-23, HA actually induced robust levels of IL-23, despite this IL-17A was inhibited by H3N2 HA and reduced by H1N1 HA. HA also induced IL-1β when combined with HKSP. These results also suggest that it is the selective suppression of IL-6 by H1N1 HA that contributes to the reduced IL-17A responses in human primary T cells. Several cytokines contribute to Th17 differentiation, however IL-23 has been most extensively studied as being key to the growth and survival of Th17 cells expressing IL-17A [56]. Clearly other cellular pathways that contribute to IL-17A need to be explored. Support for our findings is also evident in the recent reports that demonstrate that influenza HA or modified versions thereof can inhibit IL-17A and IL-6 production in mice with subsequent beneficial effects on collagen-induced arthritis [57]. Interestingly, authors did not present data for IL-23 in these studies. In addition, while HA exerts its effects independently of IL-23, it is clear from the live infection that inhibition of IL-23 is also involved. As the effects are observed within 24 hours of infection it is likely that other components of the virus are contributing directly to IL-23 suppression and these results suggest that the innate immune suppression is more extensive with the whole virus infection contributing to perhaps greater susceptibility during live influenza infection. We also observe that H3N2 virus appears to be more immunosuppressive than H1N1 virus, however the H1N1 HA is more immunosuppressive than the H3N2 HA. A study found that there is higher neuraminidase (NA) activity in H3N2 viruses than in H1N1 viruses [58], which leads to higher mortality from secondary bacterial pneumonia [59]. This may be why the H3N2 virus appears to exert a stronger level of inhibition compared to the H1N1 virus. The findings of this study are novel compared to previous studies in that the results suggest that early influenza infection may inhibit Th17 pathways in humans in the absence of Type I IFN, which may also be relevant to enhanced bacterial colonisation. Furthermore, the partial association of HA, which is a major component of IAV vaccines [60–64], with IL-17A down-regulation may have implications for the recommended use of multiple vaccines as a whole and particularly combined influenza and pneumococcal vaccines [65]. As suggested by Irish Health Service Executive (HSE) (https://www.hse.ie/eng/health/immunisation/pubinfo/flu-vaccination/faq.pdf), the current protocol for administering the influenza vaccine and pneumococcal conjugate vaccine (PCV) to children is to separate administration of vaccines by 1 week. Whereas the pneumococcal polysaccharide vaccine (PPV), which is administered to adults can be administered concurrently with the influenza vaccine, albeit at different sites. We would recommend that administration at different sites should continue to prevent any interference between influenza and pneumococcus vaccines from occurring.

## Supporting information

S1 Fig Representative graph showing individual donor cytokine concentrations for plots in Fig 1. (PDF)

S2 Fig Representative graph showing individual donor cytokine concentrations for plots in Fig 2. (PDF)

S3 Fig Representative graph showing individual donor cytokine concentrations for plots in Fig 4. (PDF)

S4 Fig Representative graph showing individual donor cytokine concentrations for plots in Fig 5. (PDF)

S5 Fig Representative graph showing individual donor cytokine concentrations for plots in Fig 6. (PDF)
