# Supplementary figures and images for: Influenza infection directly alters innate IL-23 and IL-12p70 and subsequent IL-17A and IFN-γ responses to pneumococcus in vitro in human monocytes
Source: PLoS One. 2018 Sep 7;13(9):e0203521. doi: 10.1371/journal.pone.0203521 (PMC6128554; doi:10.1371/journal.pone.0203521)

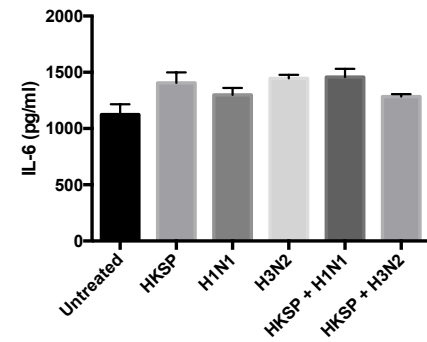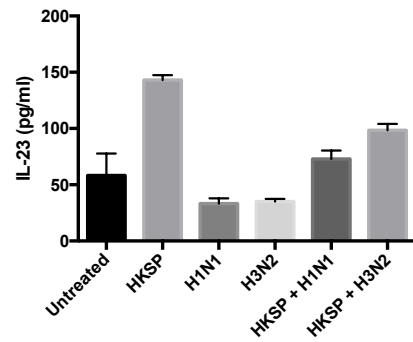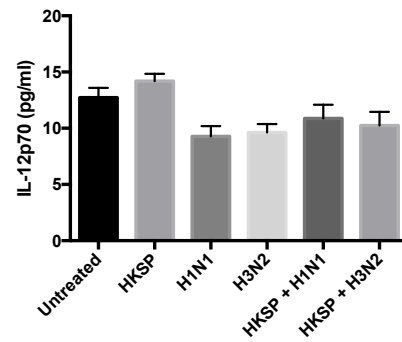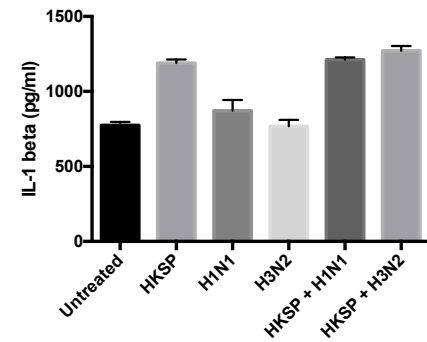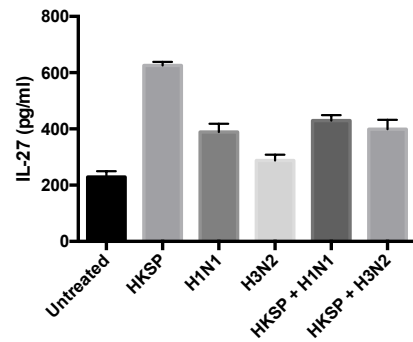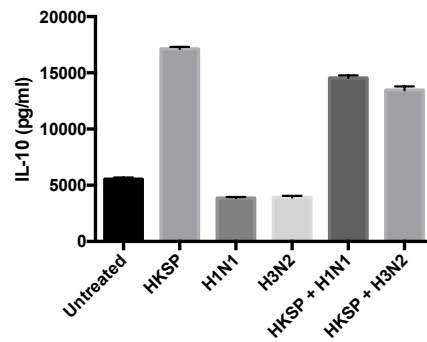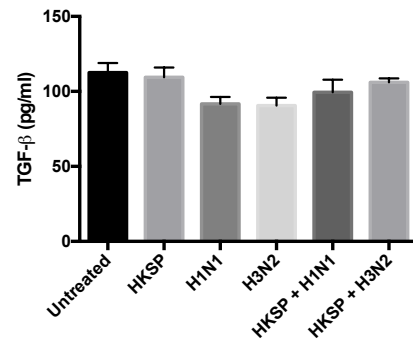

Supplement: S1 Fig — (PDF) [file pone.0203521.s001.pdf]

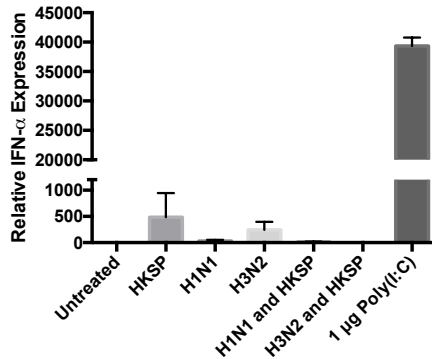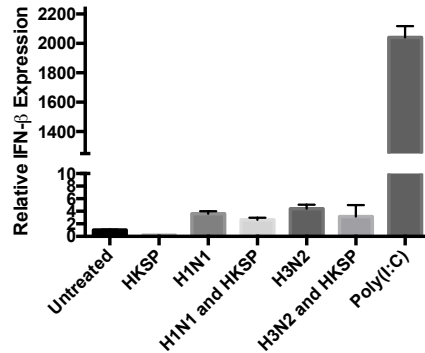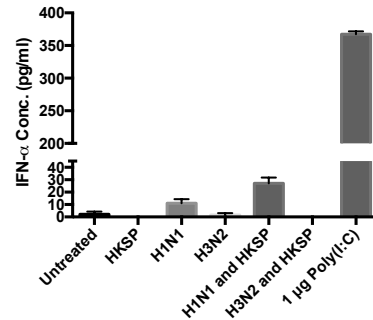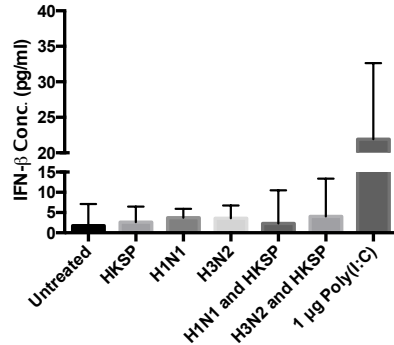

Supplement: S2 Fig — (PDF) [file pone.0203521.s002.pdf]

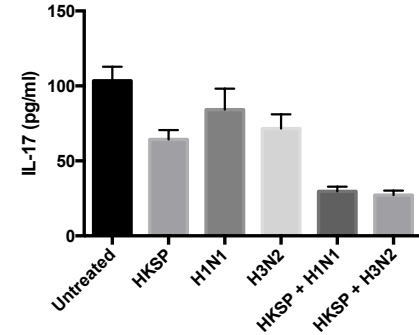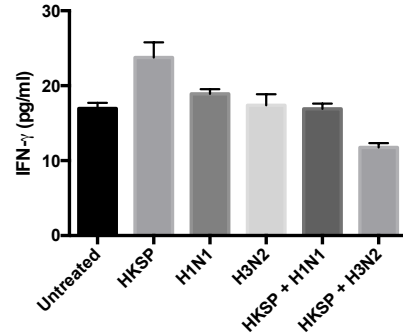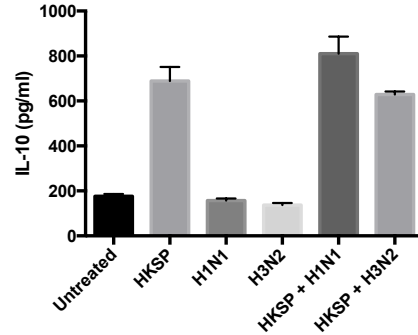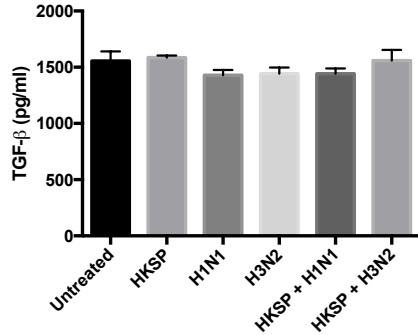

Supplement: S3 Fig — (PDF) [file pone.0203521.s003.pdf]

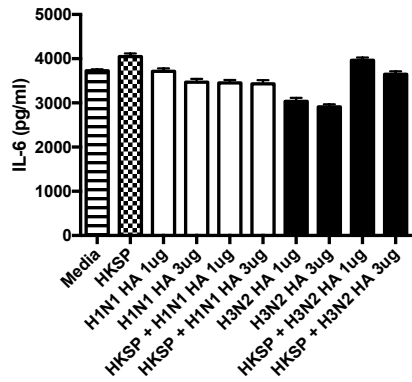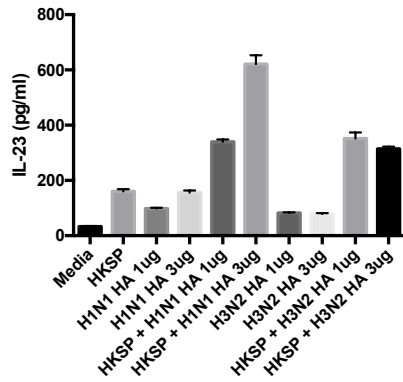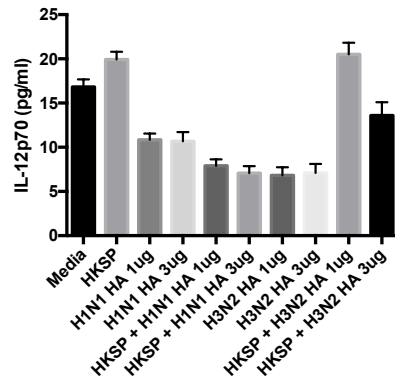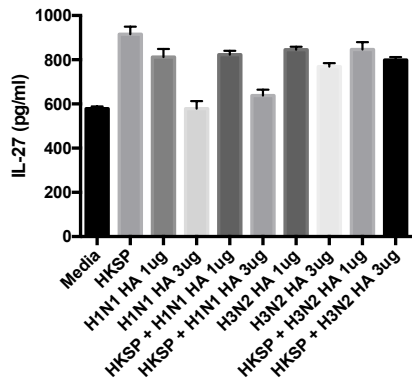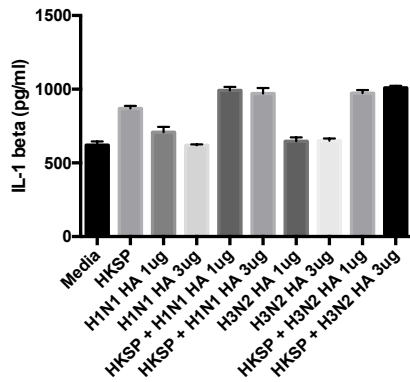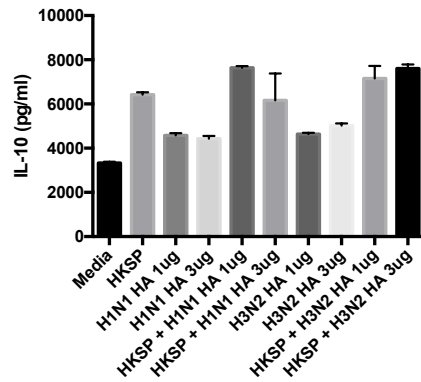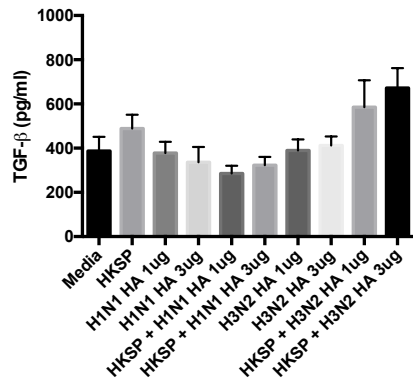

Supplement: S4 Fig — (PDF) [file pone.0203521.s004.pdf]

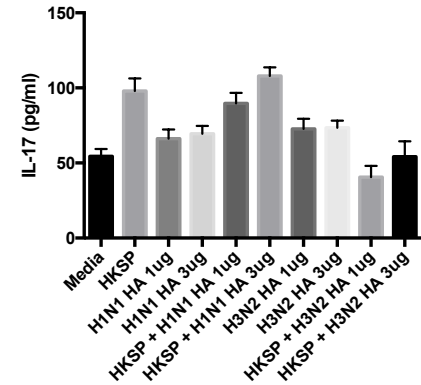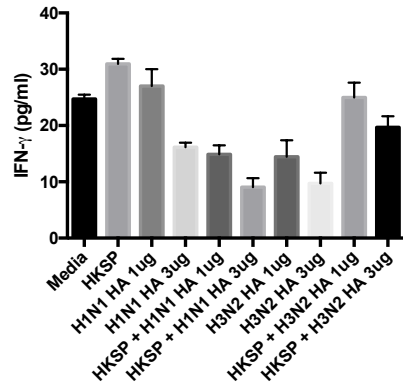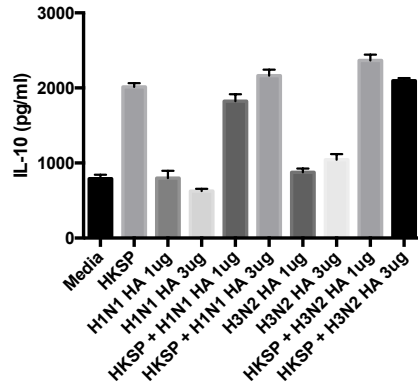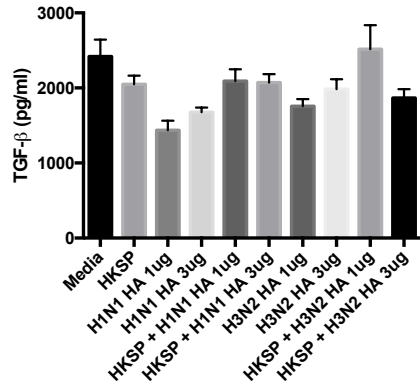

Supplement: S5 Fig — (PDF) [file pone.0203521.s005.pdf]
